# Crop Functional Genomics and Biological Breeding

**DOI:** 10.3390/plants14081258

**Published:** 2025-04-21

**Authors:** Jia Li, Jie Huang, Jiezheng Ying, Jian Zhang, Dawei Xue, Yifeng Wang

**Affiliations:** 1 State Key Laboratory of Rice Biology and Breeding, China National Rice Research Institute, Hangzhou 311400, China; lij697495@163.com (J.L.); huangjie@caas.cn (J.H.); yingjiezheng@caas.cn (J.Y.); zhangjian@caas.cn (J.Z.); 2 College of Life and Environmental Sciences, Hangzhou Normal University, Hangzhou 311121, China; dwxue@hznu.edu.cn

## 1. Introduction

Over the past two decades, the rapid development of functional genomics has gradually clarified the regulatory effects of genotypes on phenotypes. Crop breeding researchers have discovered numerous genes that influence critical traits such as yield, resistance, and quality. By targeting these genes, scientists have achieved more customised variety breeding through biological breeding. Crop functional genomics research is a vital pathway for biological breeding. Understanding the functional genomics of crops provides deep insights into the genetic mechanisms controlling key traits like yield, stress resistance, and quality improvement. This article aims to summarise the latest research and discoveries in crop functional genomics from plant journals, including studies on gene responses to stresses through whole-genome and transcriptome analyses and the identification and cloning of new genes related to crop yield, resistance, germination, and quality. This article also covers functional analyses of these genes and their applications in biological breeding.

## 2. Studying Gene Resistance Through Functional Genomics

Liu et al. conducted a genome-wide analysis of the Nramp gene family in kenaf (*Hibiscus cannabinus*), identifying 15 *HcNramp* genes. Phylogenetic analysis classified these 15 HcNramp proteins into three distinct subfamilies, with genes in the same subfamily sharing similar structures and motifs [1]. The promoters of these genes were rich in hormone-responsive and environmental stress-responsive elements. Collinearity analysis revealed 10 pairs of collinear genes between kenaf and *Arabidopsis* and another 10 pairs between kenaf and tomato. Transcriptomic data showed that all *Nramp* genes in kenaf exhibited tissue-specific expression. Under treatments with varying concentrations of cadmium (Cd) and melatonin, the expression levels of *HcNramp* genes differed across tissues and were significantly affected by Cd stress. *HcNramp3* was preferentially expressed in leaves, while *HcNramp2/12/13/15* were primarily expressed in roots. *HcNramp3/11* may regulate Cd, *HcNramp1/6/7/14* may be involved in Cd transport, and *HcNramp2/6/8/9/12* could enhance kenaf’s tolerance to Cd. These findings provide a foundation for understanding the potential role of the *Nramp* gene family in Cd stress and are significant for screening Cd-resistant genes in kenaf.

The *SAUR* (small auxin-up RNA) genes rapidly respond to auxin hormones and play key roles in various biological processes, including plant growth and abiotic stress responses. Zhu et al. conducted a genome-wide analysis in sandalwood (*Santalum album* L.), identifying 43 *SaSAUR* genes. Phylogenetic and gene structure analyses divided these genes into five groups with similar structures, with most lacking introns. Collinearity analysis revealed 14 segmental duplications and 9 tandem duplications among *SaSAUR* genes, suggesting that duplication is the primary driver of the evolutionary expansion in the *SAUR* gene family [2]. Transcriptomic data from various tissues of sandalwood showed that all 43 *SAUR* genes were expressed in leaves, with no significant differences in expression levels. *SaSAUR18* was highly expressed in roots, while *SaSAUR08* and *SaSAUR13* showed significantly higher expression in heartwood. Under salt treatments with varying concentrations, *SaSAUR04*, *SaSAUR07*, *SaSAUR08*, *SaSAUR09*, *SaSAUR18*, *SaSAUR27*, and *SaSAUR28* were upregulated considerably, indicating their potential involvement in salt stress response mechanisms. These studies contribute to a better understanding of the *SAUR* gene family, particularly its relationship with salt stress responses, and lay the groundwork for future functional analyses.

Maize lethal necrosis (MLN) poses a significant threat to food security in sub-Saharan Africa (SSA), with only a few commercial inbred lines showing tolerance. Murithi et al. performed transcriptomic analyses on four commercial maize inbred lines and one non-adapted inbred line under MLN infection [3]. They found that the gene expression patterns of different varieties did not fully align with their phenotypic resistance levels. The resistance of KS23-6 was primarily against maize chlorotic mottle virus (MCMV), while the CML lines exhibited resistance to sugarcane mosaic virus (SCMV). Differentially expressed gene (DEG) enrichment results showed that the disease resistance genes were mainly *leucine-rich repeat* (*LRR*) genes and components of the plant innate immune system, such as the RNA interference pathway and the ubiquitin–proteasome system. The expression levels of translation initiation factor *eIF4E* and elongation factor *eIF4G*, which are related to viral replication, were also closely associated with MLN viruses. These findings provide molecular-level theoretical support for MLN disease control and offer candidate gene targets for breeding MLN-resistant maize germplasm in SSA.

Serine hydroxymethyltransferase (SHMT, E.C.2.1.2.1) is a critical enzyme that catalyses serine and glycine conversion and is widely distributed in plants, animals, and microorganisms. However, systematic functional studies on SHMT gene family members in rice remain limited. Pan et al. comprehensively analysed the *SHMT* gene family [4]. Phylogenetic analysis and subcellular localisation results showed that the five *SHMT* genes in rice were distributed across three subgroups: Ia, Ib, and IIb. *OsSHMT3*, classified initially in the cytoplasm-localised Ib subgroup, was localised to chloroplasts in rice protoplasts. Pan et al.’s study also revealed a unique gene loss event in monocots, leading to the absence of the chloroplast-localised IIa subgroup of *SHMT*. Yeast two-hybrid experiments confirmed that all five OsSHMT proteins could form homodimers, with OsSHMT3 capable of forming heterodimers with other members except for OsSHMT1. Molecular docking simulations suggested that OsSHMT3 might coordinate with other members as a mobile protein. Cis-acting element predictions and expression pattern analyses indicated that *OsSHMT* family members might participate in various stress responses and hormone-regulatory pathways. In vitro experiments showed that OsSHMT1/3 exhibited enzymatic activity, while OsSHMT4/5 were inactive. Whether these proteins function as catalytic enzymes or purely regulatory elements remains to be elucidated.

Alfalfa (*Medicago sativa* L.), a crucial perennial leguminous forage crop, is widely recognised as the “King of Forages” due to its exceptional nutritional value, palatability, and adaptability. In Northeast China, where vast areas of saline–alkali land remain underutilised, investigating alfalfa’s salt tolerance is vital for developing salt-tolerant cultivars and reclaiming these marginal lands. Through a two-phase screening process of 41 alfalfa varieties, Wang et al. identified Longmu801 as a salt-tolerant cultivar and WL168 as a salt-sensitive one [5]. Transcriptomic analysis revealed that differentially expressed genes (DEGs) in both Longmu801 and WL168 under salt stress conditions showed significant enrichment in oxidative stress response, nuclear activities, and plasma membrane functions. KEGG pathway analysis further demonstrated close associations between salt tolerance and key metabolic pathways, including phenylpropanoid biosynthesis, endoplasmic reticulum protein processing, and starch and sucrose metabolism. Using weighted gene co-expression network analysis (WGCNA), researchers identified six modules related to salt tolerance and subsequently pinpointed five hub genes directly responsive to salt stress: *Msa085011*, *Msa0605650*, *Msa0397400*, *Msa1258740*, and *Msa0958830*. In conclusion, while salt-tolerant and salt-sensitive cultivars share similar growth and physiological response mechanisms under salt stress, the salt-tolerant Longmu801 exhibits superior tolerance through the enhanced accumulation of osmolytes, elevated antioxidant enzyme activities, and distinct gene expression patterns. This study provides valuable candidate genes for breeding salt-tolerant alfalfa varieties and offers scientific foundations for the sustainable utilisation of saline–alkali soils.

## 3. Functional Analysis of Plant Trait-Related Genes

The BBX protein family is highly conserved in plants, with 32 BBX genes in *Arabidopsis* and 30 in rice. BBX proteins involve diverse plant regulatory networks, ranging from seedling photomorphogenesis to stress responses. Yang et al. identified a BBX protein, OsBBX2, which is highly expressed in rice roots and exhibits diurnal rhythmic transcription [6]. Overexpressing *OsBBX2* in the *japonica* rice variety Longjing 11 (LJ11), which has weakened or lost functions of *Ghd7* and *PRR37*, delayed heading under long-day (LD) conditions. The flowering phenotype of the *bbx2* mutant was not significantly different from that of the wild type (WT), possibly due to functional redundancy among other BBX family members. In *OsBBX2*-overexpressing plants, *Ehd1*, *Hd3a*, and *RFT* expression levels were significantly reduced. Yeast two-hybrid experiments revealed that OsBBX2 interacts with Hd1 (BBX18), substantially suppressing *Hd3a* expression. OsBBX2 and Hd1 synergistically inhibit *Hd3a* expression to delay rice flowering. Meanwhile, the *bbx2* and *hd1* double mutant exhibited a late-flowering phenotype similar to *hd1*.

Research on rice grain size is crucial for breeding high-yield and high-quality rice varieties. Plant hormones are important factors regulating grain development. Aux/IAA family proteins, as core components of the auxin signalling pathway, are plant-specific transcription factors that play vital roles in regulating plant growth and development. Although *Arabidopsis* Aux/IAA family genes have been extensively studied, the functions of most members in rice remain unclear. Only two genes, *OsIAA3* and *OsIAA10*, have been reported to regulate rice grain size. Jia et al. found that the *osiaa19* mutant exhibited significantly increased grain length and thousand-grain weight, with no significant effects on agronomic traits such as plant height, tiller number, or flag leaf length [7]. However, the chalkiness of *osiaa19* grains increased, which might be related to the transient starch degradation enzyme GWD1 in source tissues. The high expression of *OsIAA19* in source tissues may indirectly cause chalkiness by affecting the carbon supply. This study provides new insights into the auxin signalling regulatory network and has significant application value for rice quality improvement.

The composition and distribution of storage substances in rice endosperm directly affect rice quality. Yang et al. identified a floury endosperm mutant, *wcr* (white-core rice), which exhibited a unique “transparent periphery-floury core” phenotype [8]. Microscopic observations revealed loosely arranged starch granules with large pores in the inner endosperm, while the outer region had densely packed starch granules. Compared to the wild type, brown rice’s total starch and amylose content showed no changes, but the levels of four protein components were significantly reduced. In milled rice (inner endosperm), total starch and amylose content decreased significantly, while albumin content nearly tripled. Through gene mapping and sequence alignment, Yang et al.’s study revealed that the *wcr* mutant phenotype was regulated by the recessive nuclear gene *OsPPDK8*, which encodes pyruvate phosphate dikinase. The mutation in *OsPPDK8* rendered it unable to catalyse pyruvate and inorganic phosphate (Pi), leading to energy deficiency, impaired internal substance transport, and developmental defects. *OsPPDK8* expression peaked at 10 days after flowering and was rapidly inactivated through threonine phosphorylation and protein degradation mechanisms. This period coincides with the critical phase of storage substance accumulation from the endosperm centre to the periphery, potentially explaining the severe floury phenotype in the inner endosperm. However, further experimental validation is required to determine whether this regulatory mechanism is accurate. This study confirms that *wcr* is a key regulator, coordinating protein synthesis and starch metabolism to regulate rice quality formation jointly.

Rice tiller number directly affects final yield and is a key trait for breeding and nitrogen-efficient cultivation. Although extensive research has been conducted, the molecular mechanisms underlying the interaction between nitrogen and plant hormones in regulating tillering remain unclear. Rimi et al. performed genetic analysis and gene mapping through population construction and BSA sequencing, revealing that the dwarf and multi-tiller phenotype of the *p47dt1* mutant was controlled by the single gene *P47DT1 (D10)* [9]. By analysing the tillering response patterns of the wild-type P47-1 and the *p47dt1* mutant under different nitrogen concentrations, Rimi et al. found that the mutation in *P47DT1* caused the loss of nitrogen response capability in rice. Observations throughout the growth cycle confirmed that P47-1 consistently had fewer tillers than *p47dt1*, indicating that *P47DT1* is a key gene regulating nitrogen response in rice tillering. Further experiments with different nitrogen forms revealed that the *P47DT1* mutation altered nitrogen allocation patterns between leaves and stems through the nitrate pathway. Transcription factor binding site predictions in the *D10* gene promoter identified numerous plant hormone-responsive elements and *Arabidopsis TCP20* transcription factor binding sites, suggesting that this site in rice might bind to *TCP19*. However, these results remain speculative and require further experimental exploration to confirm the regulatory pathways and their effects on tillering.

Rice blast is a primary global disease in rice production, causing annual yield losses of 10–50%. Disease-resistant breeding is the core solution to this problem. Jin et al. cloned a new gene, *Pigm-1*, from the double-resistant line 77009, which is allelic to the known broad-spectrum resistance gene *Pigm* [10]. Resistance spectrum analysis revealed that the two genes differed only in their resistance to the JL-37 line: *Pigm* was resistant to JL-37, while *Pigm-1* was relatively susceptible. Using *Pigm-1* donor R20-4 crossed with susceptible glutinous rice S19-118, a new blast-resistant glutinous rice material, Xiannuo 23, was developed through marker-assisted selection, showing significant field resistance. Recent studies found that *PigmR* (the protein encoded by *Pigm*) enriches cell membrane microdomains via the PIBP4-Rab5a transport machinery, activating OsRac1 to trigger reactive oxygen bursts and enhance resistance. To date, *Pigm-1* has been successfully used to develop six blast-resistant restorer lines (using Minghui 63 as the receptor), four new resistant materials (using R20 as the receptor), and five materials resistant to both blast and bacterial blight (combining *Xa23*). The *Pigm* allele has been used in molecular breeding by over 30 domestic institutions, with several varieties entering regional trials. However, the molecular mechanism of JL-37 strain-specific recognition remains unresolved. This study confirms that *Pigm-1* possesses broad-spectrum resistance comparable to *Pigm*, providing a new option for disease-resistant breeding.

## 4. Conclusions and Perspectives

This paper reviews the latest research progress in crop functional genomics and biological breeding, focusing on applying gene functional genomics in crop resistance, trait regulation, and breeding. Further functional validation of identified genes (e.g., HcNramp, *SaSAUR*, *OsSHMT*, etc.) is needed to elucidate their specific mechanisms in stress resistance and quality regulation while exploring gene interaction networks to uncover the complex mechanisms of multi-gene synergistic regulation. The molecular basis of crop trait formation can be comprehensively analysed by integrating multiple omics methods, including genomics, transcriptomics, proteomics, and metabolomics. Advanced gene-editing technologies (e.g., CRISPR-Cas9) are employed to modify key genes precisely, and high-efficiency molecular marker-assisted selection techniques are developed to breed new crop varieties with enhanced stress tolerance, high yield, and superior quality. In response to global challenges such as climate change and resource scarcity, research prioritises crop response mechanisms to abiotic stresses (e.g., salinity, drought, and heavy metal contamination) to develop more resilient varieties.

Additionally, disease resistance breeding is intensified, particularly in the mining and application of broad-spectrum resistance genes against significant diseases like rice blast. Through functional genomics and biological breeding technologies, crop yield and quality can be improved while reducing reliance on chemical fertilisers and pesticides, promoting sustainable green agriculture. Furthermore, the potential of marginal lands (e.g., saline–alkali soils) is maximised to provide new solutions for food security.

In conclusion, crop functional genomics and biological breeding hold immense potential. Future efforts should strengthen the integration of fundamental research and practical applications to provide robust scientific and technological support for sustainable agricultural development.
